# The effects of angiotensinogen gene polymorphisms on cardiovascular disease outcomes during antihypertensive treatment in the GenHAT study

**DOI:** 10.3389/fphar.2014.00210

**Published:** 2014-09-16

**Authors:** Anh N. Do, Marguerite R. Irvin, Amy I. Lynch, Steven A. Claas, Eric Boerwinkle, Barry R. Davis, Charles E. Ford, John H. Eckfeldt, Hemant K. Tiwari, Nita A. Limdi, Donna K. Arnett

**Affiliations:** ^1^Department of Epidemiology, University of Alabama at BirminghamBirmingham, AL, USA; ^2^Division of Epidemiology, School of Public Health, University of Texas Health Science Center at HoustonHouston, TX, USA; ^3^Division of Biostatistics, School of Public Health, University of Texas Health Science Center at HoustonHouston, TX, USA; ^4^Department of Laboratory Medicine and Pathology, University of MinnesotaMinneapolis, MN, USA; ^5^Department of Biostatistics, University of Alabama at BirminghamBirmingham, AL, USA; ^6^Department of Neurology, University of Alabama at BirminghamBirmingham, AL, USA

**Keywords:** *AGT* gene, antihypertensive drugs, hypertension, coronary heart disease, heart failure

## Abstract

Previous studies have reported that risk of cardiovascular morbidity and mortality substantially increases in hypertensive patients, especially among those with inadequate blood pressure control. Two common antihypertensive drug classes including thiazide diuretics and angiotensin-converting enzyme (ACE) inhibitors affect different enzymes in the renin-angiotensin-aldosterone system (RAAS). In the RAAS, angiotensinogen is converted into angiotensin II which increases blood pressure through vasoconstriction. Using a case-only design with 3448 high-risk hypertensive individuals from the Genetics of Hypertension Associated Treatment (GenHAT) study, we examined whether seven single nucleotide polymorphisms (SNPs) in the angiotensinogen gene (*AGT*) interact with three classes of antihypertensive drugs including chlorthalidone (a thiazide diuretic), lisinopril (an ACE inhibitor), and amlodipine (a calcium channel blocker) to modify the risk of incident coronary heart disease (CHD) and heart failure (HF) among Caucasian and African American participants, separately. We found no gene by treatment interactions to be statistically significant after correction for multiple testing. However, some suggestive results were found. African American participants with the minor allele of rs11122576 had over two-fold higher risk of CHD when using chlorthalidone compared to using amlodipine, or lisinopril compared to amlodipine (*p* = 0.006 and *p* = 0.01, respectively). Other marginal associations are also reported among both race groups. The findings reported here suggest that rs11122576 could contribute to future personalization of antihypertensive treatment among African Americans though more studies are needed.

## Introduction

Hypertension is one of the most common conditions in the U.S. affecting about 76.4 million (about 1 out of 3) adults in 2012, and is a large contributor to heart disease and stroke incidence and mortality. Notably, less than half of all hypertensive patients have adequate blood pressure control. In 2008, the overall death rate associated with hypertension was 18.3% (Roger et al., 2012). Additionally, treating hypertension costs the U.S. economy $131 billion annually for direct medical expenses, and another $25 billion indirectly due to the loss of productivity (Heidenreich et al., 2011). Antihypertensive pharmacogenetic research has the potential to discover genetic contributors to variability in antihypertensive response, and tailoring therapy based on an individual's genetic make-up has the potential to mitigate cardiovascular disease (CVD) outcomes among treated hypertensives.

**Figure 1:**
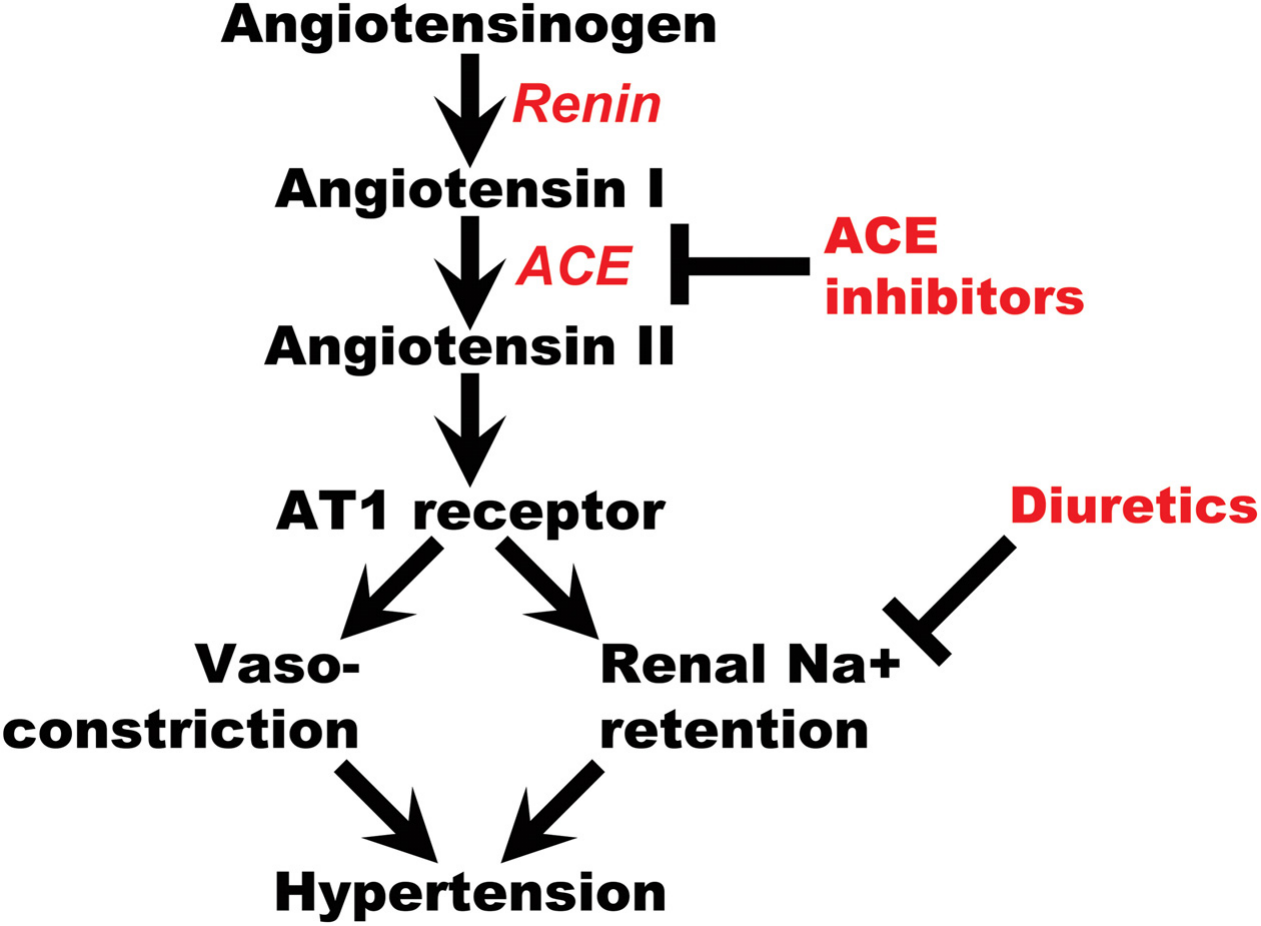
**Renin-Angiotensin pathway and the roles of ACE inhibitors, diuretics**.

The renin-angiotensin-aldosterone system (RAAS) plays a major role in maintaining salt-water balance and controlling blood pressure (Li et al., 2013). Multiple studies have reported genes belonging to this pathway are associated with hypertension and its sequelae (Jeunemaitre et al., 1992; Mahmood et al., 2003; Pasquale et al., 2005; Al-Najai et al., 2013; Li et al., 2013). Additionally, many antihypertensive drugs act to downregulate this pathway. Therefore, genes belonging to the RAAS may modify antihypertensive treatment response and risk for adverse outcomes (Figure 1). Angiotensinogen is an upstream member of the RAAS encoded by the *AGT* gene on chromosome 1. It is primarily synthesized in the liver and then cleaved by renin from the kidney to form angiotensin I. Angiotensin I is further cleaved by the angiotensin-converting enzyme (ACE) to form the biologically active form called angiotensin II. In the kidney, angiotensin II induces vessel contraction via the AT1 receptor and Na^+^ reabsorption in epithelial cells at the proximal tubules via aldosterone release (Craig and Stitzel, 2003). Among the three most widely used antihypertensive drug classes (diuretics, ACE inhibitors, and calcium channel blockers), ACE inhibitors directly affect the RAAS through blocking the conversion of angiotensin I to angiotensin II. Thiazide diuretics can acutely activate RAAS through the inhibition of Na^+^ reabsorption and reduction of plasma volume (Sica, 2004; Duarte and Cooper-DeHoff, 2010). Polymorphisms in the *AGT* gene have been found to be associated with CVD, especially coronary heart disease (CHD), and heart failure (HF) (Chen et al., 2012; Li et al., 2013). Therefore, we hypothesize that CHD and HF risk among hypertensive patients treated with these drugs may be modified by variations in the *AGT* gene. Polymorphisms in the *AGT* gene may affect this risk directly or through downstream effects on other members of the RAAS pathway.

In the current study, we evaluated whether seven SNPs in the *AGT* gene affect risk of CHD and HF in high-risk hypertensive individuals randomized to one of three classes of antihypertensive treatment as part of the Genetics of Hypertension Associated Treatment Study (GenHAT) using a case-only design (Arnett et al., 2002).

## Materials and methods

### Study population

The GenHAT study (*N* = 39,114) was initiated to evaluate if several genes associated with hypertension modify the risk of CHD and other CVD outcomes in patients taking different anti-hypertensive drug classes (Arnett et al., 2002). GenHAT is an ancillary study of the Antihypertensive and Lipid Lowering Treatment to Prevent Heart Attack Trial (ALLHAT), a randomized, double blind, multicenter clinical trial with 42,418 high-risk hypertensive persons 55 years of age or older. Participants were randomized into four groups defined by the class of antihypertensive medication they were assigned to, including chlorthalidone, lisinopril, amlodipine, and doxazosin (an α-adrenergic blocker), in a ratio of 1.7:1:1:1, respectively. Due to the early discontinuation of the doxazosin arm owing to a significant (25%) increase in the incidence of CVD compared to the chlorthalidone arm, we have not included the doxazosin arm in these analyses (ALLHAT Collaborative Research Group, 2000). Participants randomized to chlorthalidone, lisinopril, or amlodipine were followed for an average of 4.9 years. For more details regarding study design and methodology related to GenHAT and ALLHAT, see Arnett et al. (2002) and Davis et al. (1996). In the case-only phase of GenHAT, 9336 participants from three treatment groups affected by fatal CHD, non-fatal myocardial infarction (MI), stroke, HF, coronary revascularization, angina, peripheral arterial disease, end-stage renal disease, or all-cause death, were genotyped for variants in several hypertension-associated genes, including the *AGT* gene.

The results presented here focus on two outcomes, namely, CHD and HF. The primary outcome for both ALLHAT and GenHAT was CHD, defined as fatal CHD or non-fatal MI (*N* = 2253). The secondary outcome for this study included HF (treated, fatal, or hospitalized) (*N* = 1728). Outcomes were reported by the clinical investigators and validated through the review of death certificates and hospitalization records. For a more detailed description of ALLHAT outcomes, see Davis et al. (1996).

This research was approved by the institutional review boards at the University of Alabama at Birmingham, the University of Texas Health Science Center, and the University of Minnesota. Informed consent was collected from all participants in ALLHAT.

### Genotyping methods

The DNA of participants from GenHAT was extracted from stored blood using Gentra Purgene Kits (Minneapolis, MN) and 600 loci that were potential candidates for blood pressure regulation or CVD were genotyped by Illumina (San Diego, CA, USA) technology. The amplification success rate was 97%, and the reproducibility was found to be 99.99%. Tag SNPs were selected using the Haploview Program (http://www.broad.mit.edu/mpg/haploview/) and based on two ethnic sources: the Caucasian (CEU) and Yoruban (YRI) population from the International HapMap project (http://www.hapmap.org/). The 10 *AGT* polymorphisms genotyped in GenHAT were rs7079, rs5051, rs5041, rs3789678, rs34829218, rs2493133, rs2493129, rs2478544, rs11568032, rs11122576. Three *AGT* polymorphisms (rs5041, rs34829218, rs11568032) with minor allele frequency <5% were not included in the analyses presented.

### Statistical methods

**Figure 2:**
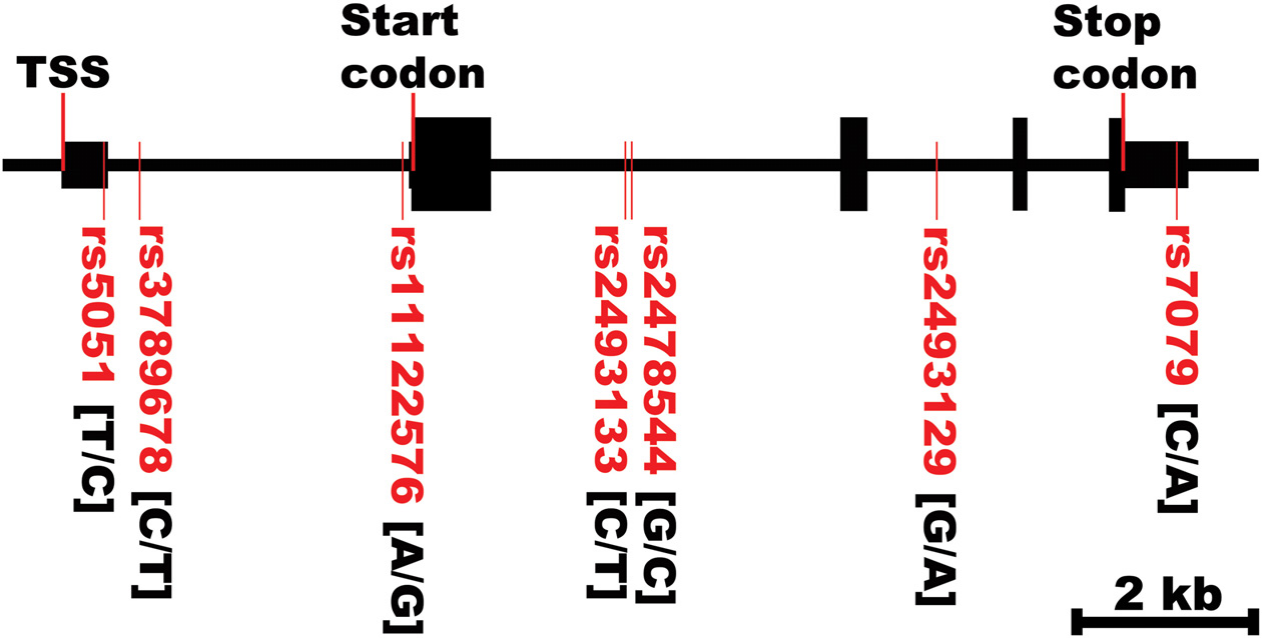
***AGT* gene structure and SNPs locations**. TSS, transcriptional start site. The first bases in the brackets are the common alleles.

To test for baseline differences between treatment groups, we used ANOVA for continuous variables and chi-square tests or Fisher's exact test (i.e., for any cell size < 10 observations) for categorical variables. Principal component analysis (PCA) of 64 ancestry informative markers (AIMs) was used to inform genetically determined Caucasian and African American race (Lynch et al., 2012). Each of the seven *AGT* SNPs was tested for Hardy Weinberg Equilibrium (HWE) stratified by genetically defined race, which will be referred to as race from here on for simplicity. The linkage disequilibrium (LD) between the seven *AGT* SNPs was analyzed using HAPLOVIEW (Figure 2) (Copyright © 2003–2006 Broad Institute of MIT and Harvard). Logistic regression was used to test for gene-by-treatment effects among the case groups, with treatment group modeled as the dependent variable and genotype as the independent variable. Three separate comparisons (chlorthalidone vs. amlodipine, chlorthalidone vs. lisinopril, and lisinopril vs. amlodipine) were conducted to test the interaction of genes and treatments. The additive genetic model was tested where the common allele homozygote was treated as the reference group. The dominant genetic model was used for SNPs which have any cell size less than 10 observations. A *p*-value of 0.05 was considered as suggestive evidence for an association. A Bonferroni correction was used to adjust for multiple testing within each outcome and race: specifically, we considered six independent SNPs (rs5051 and rs2493133 were determined to be in strong LD, described below) and three treatment comparisons; therefore, α was adjusted to 0.05/18 = 0.003. SAS version 9.3 (SAS Institute Inc., Cary, NC, USA) was used for all analyses. Using Quanto V. 1.2 software (Gauderman and Morrison, 2009) for the case-only design with allele frequency is 0.05 or greater, and type 1 error rate set to 0.003, we have 81% power to detect an interaction odds ratio of 1.80 or greater for Caucasians and 80% power to detect an interaction odds ratio of 2.2 or greater for African-Americans.

## Results

Table 1 shows the baseline characteristics of 3448 subjects included in this analysis. There were no significant differences in the baseline characteristics between three medication arms. Table 2 presents allele frequencies for the seven *AGT* variants by ethnic subgroups. The allelic frequency of the seven *AGT* variants was not significantly different between three medication arms in either race group except for rs2493129 among Caucasians. The minor allele frequencies for rs5051 and rs2493331 differed considerably between African Americans and Caucasians, but minor allele frequencies were comparable in both races for all other SNPs.

**Table 1:** **Baseline characteristics for case-only participants (*n* = 3448) by treatment group, mean (SD) unless otherwise noted**.

**Characteristics**	**Chlorthalidone**	**Amlodipine**	**Lisinopril**	***P*-value**
Sample size (*n*, %)	1511 (43.8)	1004 (29.1)	933 (27.1)	
Age, mean	69.6 (8.1)	69.1 (8.0)	69.8 (8.1)	0.13
Caucasians^a^, *n* (col%)	1000 (60.6)	649 (58.5)	597 (58.5)	0.93
African American^a^, *n* (col%)	511 (31.0)	355 (32.0)	336 (32.9)	
Women, *n* (col %)	605 (40.0)	403 (40.1)	375 (40.2)	0.99
Previous antihypertensive treatment, *n* (col%)	1385 (91.7)	942 (93.8)	862 (92.4)	0.13
Baseline SBP in mmHg, mean (*SD*)	147.4 (16.2)	146.9 (16.5)	147.9 (16.1)	0.40
Baseline DBP in mmHg, mean (SD)	82.4 (10.3)	82.0 (10.5)	82.2 (11.0)	0.70
**ELIGIBILITY RISK FACTORS**				
Current cigarette smoker, *n* (col %)	319 (21.1)	202 (20.1)	210 (22.5)	0.66
History of MI or stroke, *n* (%)	563 (37.3)	350 (34.9)	323 (34.6)	0.31
History of CABG, *n* (%)	344 (22.8)	211 (21.0)	215 (23.0)	0.49
Other atherosclerotic CVD, *n* (%)	449 (29.7)	313 (31.2)	271 (29.1)	0.57
Major ST depression/T-wave inversion, *n* (%)	177 (11.8)	135 (13.5)	104 (11.2)	0.25
Type 2 diabetes, *n* (%)	654 (43.3)	444 (44.2)	400 (42.9)	0.82
HDL-C <35 mg/dL, *n* (%)	191 (12.6)	108 (10.8)	92 (9.9)	0.09
LVH by electrocardiogram, *n* (%)	256 (16.9)	177 (17.6)	172 (18.4)	0.64
Body mass index, mean (*SD*), kg/m^2^	29.9 (6.6)	30.2 (6.6)	29.6 (6.4)	0.11
Current Aspirin use, *n* (%)	668 (44.2)	443 (44.1)	424 (45.4)	0.67
Current Estrogen supplementation,*n* (%)	82 (13.6)	61 (15.1)	42 (11.2)	0.35
Fasting glucose, mg/dL	132.9 (63.8)	130.4 (63.5)	127.7 (60.8)	0.23
Creatinine, mg/dL	1.1 (0.4)	1.1 (0.3)	1.1 (0.3)	0.62
Total cholesterol, mg/dL	218.2 (46.8)	216.9 (44.9)	216.5 (44.0)	0.64
HDL cholesterol, mg/dL	44.5 (14.7)	45.5 (14.5)	44.9 (14.1)	0.24
LDL cholesterol, mg/dL	138.3 (37.3)	136.9 (39.1)	138.3 (38.4)	0.64
Fasting triglycerides, mg/dL	188.1 (164.5)	178.6 (144.9)	177.9 (137.5)	0.25
Potassium, mmol/L	4.4 (0.5)	4.3 (0.5)	4.3 (0.5)	0.74

*SBP, systolic blood pressure; DBP, diastolic blood pressure; MI, myocardial infraction; CABG, coronary artery bypass grafting; CVD, cardiovascular disease; CHD, coronary heart disease; HDL-C, HDL cholesterol; LVH, left ventricular hypertrophy*.

*Test of differences between genotype groups: ANOVA for continuous variables, chi-square for categorical variables*.

a *Race is genetically determined*.

**Table 2A:** **Alleles frequency of 7 AGT variants and *p*-values corresponding to the test of allele frequencies differences among treatments for case-only participants among Caucasians (*n* = 2246)**.

**AGT variants**		**Chlorthalidone**	**Amlodipine**	**Lisinopril**	***P*-value**
rs7079	CC	437 (45.5%)	297 (47.7%)	252 (44.8%)	0.33
	AC	402 (41.9%)	258 (41.4%)	255 (45.4%)	
	AA	121 (12.6%)	68 (10.9%)	55 (9.8%)	
rs5051	TT	193 (19.8%)	120 (19.1%)	108 (18.7%)	0.22
	TC	435 (44.5%)	316 (50.2%)	270 (46.6%)	
	CC	349 (35.7%)	194 (30.8%)	201 (34.7%)	
rs3789678	CC	779 (79.3%)	494 (77.8%)	462 (79.5%)	0.89
	TC	188 (19.1%)	132 (20.8%)	109 (18.8%)	
	TT	16 (1.6%)	9 (1.4%)	10 (1.7%)	
rs2493133	CC	194 (20.2%)	117 (18.7%)	106 (18.5%)	0.24
	TC	423 (43.9%)	311 (49.7%)	268 (46.8%)	
	TT	346 (35.9%)	198 (31.6%)	199 (34.7%)	
rs2493129	GG	941 (95.1%)	587 (91.7%)	551 (93.1%)	0.01
	AG	46 (4.7%)	53 (8.3%)	40 (6.8%)	
	AA	3 (0.3%)	0 (0%)	1 (0.2%)	
rs2478544	GG	642 (68.4%)	396 (65.7%)	386 (68.7%)	0.36
	GC	263 (28%)	191 (31.7%)	154 (27.4%)	
	CC	34 (3.6%)	16 (2.7%)	22 (3.9%)	
rs11122576	AA	863 (87.6%)	543 (84.5%)	493 (83.3%)	0.07
	AG	112 (11.4%)	96 (14.9%)	91 (15.4%)	
	GG	10 (1%)	4 (0.6%)	8 (1.4%)	

*Test of differences between genotype groups: chi-square and Fisher's exact tests. Fisher's exact test was used for SNP which has any cell size less than 10 observations in frequency table*.

**Table 2B:** **Alleles frequency of 7 AGT variants and *p*-values corresponding to the test of allele frequencies differences among treatments for case-only participants among African Americans (*n* = 1202)**.

**AGT variants**		**Chlorthalidone**	**Amlodipine**	**Lisinopril**	***P*-value**
rs7079	CC	401 (80%)	296 (85.6%)	281 (85.2%)	0.11
	AC	91 (18.2%)	47 (13.6%)	42 (12.7%)	
	AA	9 (1.8%)	3 (0.9%)	7 (2.1%)	
rs5051	TT	353 (70.5%)	258 (73.7%)	244 (73.7%)	0.11
	TC	128 (25.6%)	88 (25.1%)	76 (23%)	
	CC	20 (4%)	4 (1.1%)	11 (3.3%)	
rs3789678	CC	316 (62.8%)	221 (64.4%)	196 (59.2%)	0.43
	TC	153 (30.4%)	106 (30.9%)	116 (35.1%)	
	TT	34 (6.8%)	16 (4.7%)	19 (5.7%)	
rs2493133	CC	319 (64.2%)	229 (67.6%)	222 (68.3%)	0.11
	TC	155 (31.2%)	103 (30.4%)	86 (26.5%)	
	TT	23 (4.6%)	7 (2.1%)	17 (5.2%)	
rs2493129	GG	437 (86.2%)	296 (84.8%)	277 (83.4%)	0.83
	AG	67 (13.2%)	51 (14.6%)	52 (15.7%)	
	AA	3 (0.6%)	2 (0.6%)	3 (0.9%)	
rs2478544	GG	150 (31.9%)	88 (27.2%)	107 (34.6%)	0.26
	GC	222 (47.2%)	170 (52.5%)	137 (44.3%)	
	CC	98 (20.9%)	66 (20.4%)	65 (21%)	
rs11122576	AA	452 (89.2%)	328 (94%)	295 (88.3%)	0.05
	AG	52 (10.3%)	19 (5.4%)	37 (11.1%)	
	GG	3 (0.6%)	2 (0.6%)	2 (0.6%)	

*Test of differences between genotype groups: chi-square and Fisher's exact tests. Fisher's exact test was used for SNP which has any cell size less than 10 observations in frequency table*.

Many of the variants were not in HWE in the either race group, possibly due to the case-only design as alleles contributing to “case” status may not be random. In the Caucasian group, rs7079 (*p* = 0.14) and rs2478544 (*p* = 0.09) were in HWE but five SNPs were not (rs5051, rs3789678, rs2493133, rs2493129, rs11122576, *p* < 0.05). For the African-American group, five SNPs were in HWE (*p* > 0.05) but rs7079 (*p* = 0.006) and rs3789678 (*p* = 0.003) were not.

**Figure 3:**
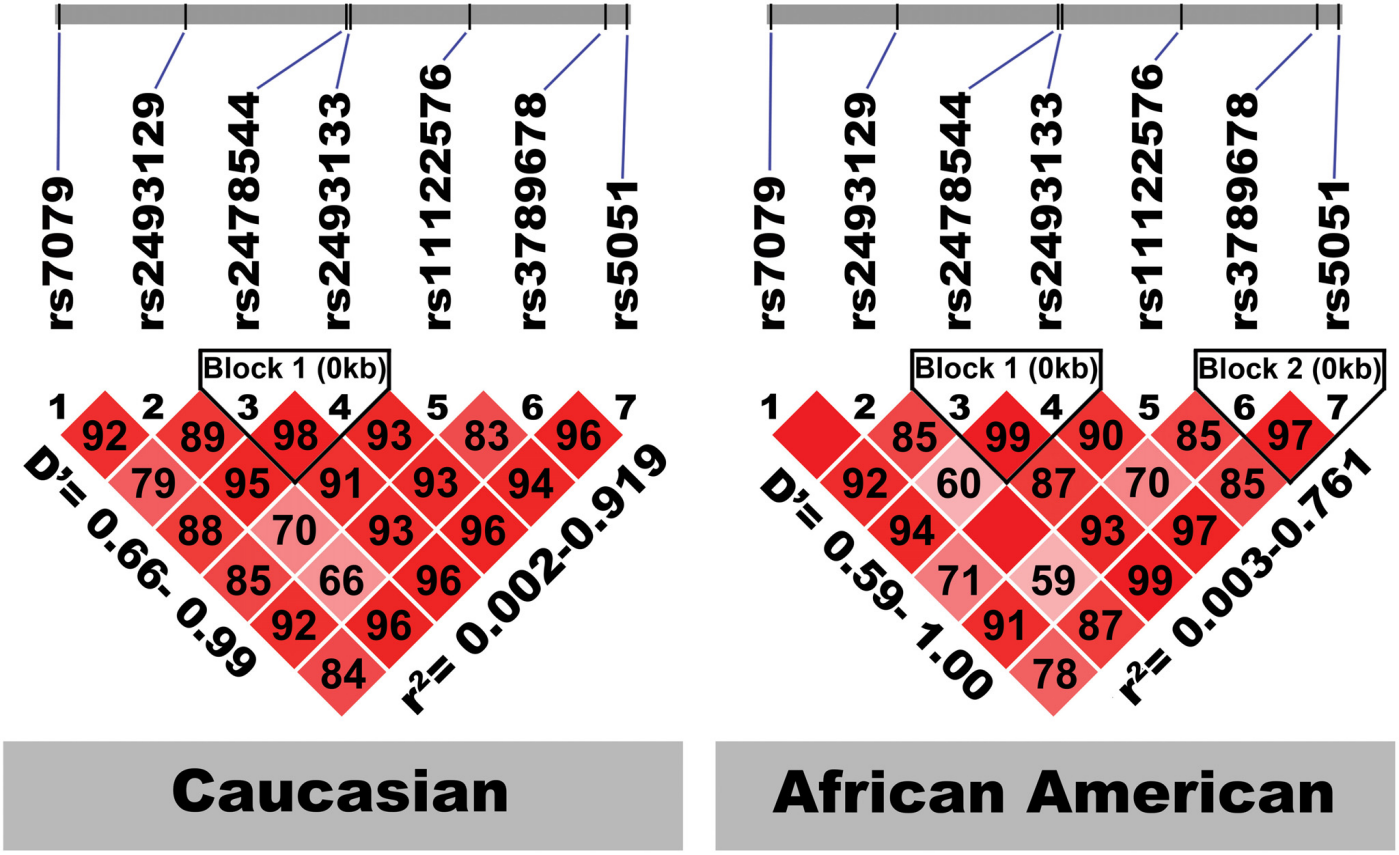
**Linkage disequilibrium structure of the seven studied *AGT* SNPs by race-specific group**. The SNPs are shown sequentially as they appear on the chromosome (not to scale). *D*', coefficient of linkage disequilibrium; *r*, regression coefficient of linkage disequilibrium.

Figure 2 shows the location of the seven SNPs in the *AGT* gene considered in this study. Five of the seven polymorphisms (rs3789678, rs11122576, rs2493133, rs2478544, rs2493129) were intronic and two (rs5051, rs7079) were in untranslated regions. Two SNPs were in tight LD (i.e., rs5051 and rs2493133) in both race groups, as calculated using HAPLOVIEW (Figure 3). The correlation coefficients for rs5051-rs2493133 were similar in Caucasians (*r*^2^ = 0.919) and African-Americans (*r*^2^ = 0.761).

The race-specific analysis includes 1500 and 1094 cases of CHD and HF, respectively, for the Caucasian group, and 753 and 634 cases of CHD and HF, respectively, for the African-Americans. Tables 3A,B demonstrate the results of the gene by treatment interaction analyses for the seven *AGT* SNPs. Overall there was little consistency by race. Among African-Americans, those with the minor allele (A) of rs7079 were 90% more likely to have CHD when using chlorthalidone compared to those using amlodipine (*p* = 0.01). Additionally, African-American participants with the minor allele (G) of rs11122576 were over two times as likely to have CHD when using chlorthalidone compared to amlodipine (OR = 2.5, *p* = 0.006) and using lisinopril compared to amlodipine (OR = 2.6, *p* = 0.01). Among Caucasian participants, those with the minor allele (G) of rs11122576 were 40% less likely to have CHD when using chlorthalidone compared to using amlodipine (OR = 0.6, *p* = 0.01), and were 30% less likely to have CHD when using chlorthalidone compared to using lisinopril (OR = 0.7, *p* = 0.03).

**Table 3A:** **Genotype-by-treatment interaction results for genetic model (additive and dominant) among Caucasians**.

**Out-come**	***AGT* variant**		**Number of cases (col %)**			**ORs and *p*-values for genetic model, Caucasians**		
			**CHL**	**AML**	**LIS**	**CHL vs. AML**	**CHL vs. LIS**	**LIS vs. AML**
CHD (*n* = 1500)	rs7079	CC	293 (43.9%)	191 (47.6%)	163 (44.2%)	*p* = 0.13; OR = 1.2; CI(0.96–1.4)	*p* = 0.62; OR = 1.0; CI(0.9–1.3)	*p* = 0.36; OR = 1.1; CI(0.9–1.4)
		AC	289 (43.3%)	170 (42.4%)	166 (45.0%)			
		AA	85 (12.7%)	40 (10.0%)	40 (10.8%)			
	rs5051	TT	135 (19.9%)	75 (18.7%)	65 (17.1%)	*p* = 0.26; OR = 1.1; CI(0.9–1.3)	*p* = 0.49; OR = 0.9; CI(0.8–1.1)	*p* = 0.10; OR = 1.2; CI(1.0–1.4)
		TC	296 (43.7%)	205 (51.1%)	175 (46.1%)			
		CC	247 (36.4%)	121 (30.2%)	140 (36.8%)			
	rs3789678	CC	542 (79.7%)	315 (78.0%)	312 (80.6%)	*p* = 0.50; OR = 0.9; CI(0.7–1.2)	*p* = 0.72; OR = 1.0; CI(0.8–1.4)	*p* = 0.36; OR = 0.9; CI(0.6–1.2)
		TC&CC	138 (20.3%)	89 (22%)	75 (19.4%)			
	rs2493133	CC	136 (20.4%)	76 (19.0%)	63 (16.9%)	*p* = 0.43; OR = 1.1; CI(0.9–1.3)	*p* = 0.28; OR = 0.9; CI(0.8–1.1)	*p* = 0.09; OR = 1.2; CI(1.0–1.5)
		TC	291 (43.6%)	201 (50.1%)	170 (45.6%)			
		TT	240 (36.0%)	124 (30.9%)	140 (37.5%)			
	rs2493129	GG	652 (95.2%)	378 (92.2%)	363 (93.3%)	*p* = 0.04; OR = 0.6; CI(0.4–1.0)	*p* = 0.20; OR = 0.7; CI(0.4–1.2)	*p* = 0.54; OR = 0.8; CI(0.5–1.4)
		AG&AA	33 (4.8%)	32 (7.8%)	26 (6.7%)			
	rs2478544	GG	452 (69.1%)	258 (68.1%)	254 (68.7%)	*p* = 0.73; OR = 1.0; CI(0.7–1.3)	*p* = 0.88; OR = 1.0; CI(0.7–1.3)	*p* = 0.87; OR = 1.0; CI(0.7–1.3)
		GC&CC	202 (30.9%)	121 (31.9%)	116 (31.3%)			
	rs11122576	AA	603 (88.7%)	342 (83.4%)	328 (84.1%)	*p* = 0.01; OR = 0.6; CI(0.5–0.9)	*p* = 0.03; OR = 0.7; CI(0.5–1.0)	*p* = 0.79; OR = 1.0; CI(0.7–1.4)
		AG&GG	77 (11.3%)	68 (16.6%)	62 (15.9%)			
HF (*n* = 1094)	rs7079	CC	208 (47.8%)	160 (48.0%)	121 (43.8%)	*p* = 0.90; OR = 1.0; CI(0.8–1.2)	*p* = 0.98; OR = 1.0; CI(0.8–1.3)	*p* = 0.92; OR = 1.0; CI(0.8–1.3)
		AC	173 (40.0%)	133 (40.0%)	132 (47.8%)			
		AA	54 (12.4%)	40 (12.0%)	23 (8.3%)			
	rs5051	TT	90 (20.4%)	68 (20.0%)	56 (19.5%)	*p* = 0.50; OR = 1.1; CI(0.9–1.3)	*p* = 0.84; OR = 1.0; CI(0.8–1.3)	*p* = 0.67; OR = 1.0; CI(0.8–1.3)
		TC	197 (44.6%)	166 (48.8%)	136 (47.4%)			
		CC	155 (35.1%)	106 (31.2%)	95 (33.1%)			
	rs3789678	CC	351 (78.9%)	266 (77.8%)	222 (78.7%)	*p* = 0.71; OR = 0.9; CI(0.7–1.3)	*p* = 0.96; OR = 1.0; CI(0.7–1.4)	*p* = 0.78; OR = 0.9; CI(0.6–1.4)
		TC&TT	94 (21.1%)	76 (22.3%)	60 (21.3%)			
	rs2493133	CC	87 (20.0%)	64 (18.9%)	56 (19.8%)	*p* = 0.56; OR = 1.1; CI(0.9–1.3)	*p* = 0.55; OR = 1.1; CI(0.9–1.3)	*p* = 0.96; OR = 1.0; CI(0.8–1.2)
		TC	192 (44.0%)	166 (49.1%)	135 (47.7%)			
		TT	157 (36.0%)	108 (32.0%)	92 (32.5%)			
	rs2493129	GG	429 (95.6%)	314 (91.3%)	269 (92.4%)	*p* = 0.02; OR = 0.5; CI(0.3–0.9)	*p* = 0.08; OR = 0.6; CI(0.3–1.1)	*p* = 0.60; OR = 0.9; CI(0.5–1.5)
		AG&AA	20 (4.4%)	30 (8.7%)	22 (7.6%)			
	rs2478544	GG	284 (67.6%)	212 (64.1%)	187 (68%)	*p* = 0.31; OR = 0.9; CI(0.6–1.2)	*p* = 0.92; OR = 1.0; CI(0.7–1.4)	*p* = 0.31; OR = 0.8; CI(0.6–1.2)
		GC&CC	136 (32.4%)	119 (35.9%)	88 (32.1%)			
	rs11122576	AA	384 (85.9%)	295 (85.3%)	241 (83.1%)	*p* = 0.80; OR = 0.9; CI(0.6–1.4)	*p* = 0.30; OR = 0.8; CI(0.5–1.2)	*p* = 0.46; OR = 1.2; CI(0.8–1.8)
		AG&GG	63 (14.1%)	51 (14.8%)	49 (16.9%)			

*CHL, chlorthalidone; AML, amlodipine; LIS, lisinopril; CHD, coronary heart disease; HF, heart failure*.

*Dominant model was used for SNP which has any cell size less than 10 observations by collapsing minor allele homozygotes with heterozygotes which was compared to common allele homozygotes. Additive model coded common homozygotes as 0, heterozygotes as 1; rare homozygotes as 2; and assumed linearity (1-degree of freedom test)*.

**Table 3B:** **Genotype-by-treatment interaction results for genetic model (additive and dominant) among African Americans**.

**Out-come**	***AGT* variant**		**Number of cases (col %)**			**ORs and p-values for genetic model, African Americans**		
			**CHL**	**AML**	**LIS**	**CHL vs. AML**	**CHL vs. LIS**	**LIS vs. AML**
CHD (*n* = 753)	rs7079	CC	259 (78.3%)	175 (87.1%)	175 (85.0%)	*p* = 0.01; OR = 1.9; CI(1.1–3.0)	*p* = 0.06; OR = 1.6; CI (1.0–2.5)	*p* = 0.54; OR = 1.2; CI(0.7–2.1)
		AC&AA	72 (21.7%)	26 (12.9%)	31 (15%)			
	rs5051	TT	237 (71.6%)	150 (73.2%)	162 (78.3%)	*p* = 0.69; OR = 1.1; CI(0.7–1.6)	*p* = 0.09; OR = 1.4; CI(1.0–2.1)	*p* = 0.23; OR = 0.8; CI(0.5–1.2)
		TC&CC	94 (28.4%)	55 (26.8%)	45 (21.7%)			
	rs3789678	CC	215 (64.6%)	139 (69.9%)	126 (60.9%)	*p* = 0.21; OR = 1.3; CI(0.9–1.9)	*p* = 0.39; OR = 0.9; CI(0.6–1.2)	*p* = 0.06; OR = 1.5; CI(1.0–2.2)
		TC&TT	118 (35.4%)	60 (30.1%)	81 (39.1%)			
	rs2493133	CC	211 (64.5%)	134 (68.7%)	145 (71.1%)	*p* = 0.33; OR = 1.2; CI(0.8–1.8)	*p* = 0.12; OR = 1.4; CI(0.9–2.0)	*p* = 0.61; OR = 0.9; CI(0.6–1.4)
		TC&TT	116 (35.5%)	61 (31.3%)	59 (28.9%)			
	rs2493129	GG	288 (86.0%)	164 (82.4%)	180 (86.5%)	*p* = 0.27; OR = 0.8; CI(0.5–1.2)	*p* = 0.85; OR = 1.0; CI(0.6–1.7)	*p* = 0.25; OR = 0.7; CI(0.4–1.3)
		AG&AA	47 (14%)	35 (17.6%)	28 (13.5%)			
	rs2478544	GG	96 (31.3%)	48 (25.7%)	63 (32.1%)	*p* = 0.31; OR = 0.9; CI(0.7–1.1)	*p* = 0.97; OR = 1.0; CI(0.8–1.3)	*p* = 0.34; OR = 0.9; CI(0.7–1.2)
		GC	144 (47.0%)	96 (51.3%)	89 (45.4%)			
		CC	67 (21.8%)	43 (23.0%)	44 (22.5%)			
	rs11122576	AA	294 (87.5%)	191 (94.6%)	181 (87.0%)	*p* = 0.006; OR = 2.5; CI(1.2–4.9)	*p* = 0.87; OR = 1.0; CI(0.6–1.6)	*p* = 0.01; OR = 2.6; CI(1.2–5.4)
		AG&GG	42 (12.5%)	11 (5.5%)	27 (13%)			
HF (*n* = 634)	rs7079	CC	190 (80.9%)	181 (86.2%)	154 (86.0%)	*p* = 0.13; OR = 1.5; CI(0.9–2.5)	*p* = 0.16; OR = 1.5; CI(0.9–2.5)	*p* = 0.96; OR = 1.0; CI(0.6–1.8)
		AC&AA	45 (19.1%)	29 (13.9%)	25 (14%)			
	rs5051	TT	157 (67.1%)	160 (76.2%)	125 (69.8%)	*p* = 0.03; OR = 1.6; CI(1.0–2.4)	*p* = 0.55; OR = 1.1; CI(0.7–1.7)	*p* = 0.16; OR = 1.4; CI(0.9–2.2)
		TC&CC	77 (32.9%)	50 (23.8%)	54 (30.2%)			
	rs3789678	CC	147 (62.8%)	124 (60.5%)	100 (55.9%)	*p* = 0.59; OR = 0.9; CI(0.7–1.3)	*p* = 0.16; OR = 0.8; CI(0.6–1.1)	*p* = 0.38; OR = 1.2; CI(0.8–1.6)
		TC	76 (32.5%)	70 (34.2%)	68 (38.0%)			
		TT	11 (4.7%)	11 (5.4%)	11 (6.2%)			
	rs2493133	CC	145 (62.5%)	142 (69.3%)	117 (66.5%)	*p* = 0.14; OR = 1.4; CI(0.9–2.0)	*p* = 0.41; OR = 1.2; CI(0.8–1.8)	*p* = 0.56; OR = 1.1; CI(0.7–1.8)
		TC&TT	87 (37.5%)	63 (30.7%)	59 (33.5%)			
	rs2493129	GG	207 (87.7%)	189 (89.2%)	143 (80.3%)	*p* = 0.64; OR = 1.2; CI(0.6–2.1)	*p* = 0.04; OR = 0.6; CI(0.3–1.0)	*p* = 0.02; OR = 2.0; CI(1.1–3.6)
		AG&AA	29 (12.3%)	23 (10.9%)	35 (19.7%)			
	rs2478544	GG	72 (32.6%)	56 (28.4%)	63 (38.7%)	*p* = 0.56; OR = 0.9; CI(0.7–1.2)	*p* = 0.35; OR = 1.1; CI(0.9–1.5)	*p* = 0.14; OR = 0.8; CI(0.6–1.1)
		GC	105 (47.5%)	102 (51.8%)	69 (42.3%)			
		CC	44 (20.0%)	39 (19.8%)	31 (19.0%)			
	rs11122576	AA	214 (91.1%)	196 (92.9%)	161 (89.0%)	*p* = 0.48; OR = 1.3; CI(0.6–2.6)	*p* = 0.47; OR = 0.8; CI(0.4–1.5)	*p* = 0.18; OR = 1.6; CI(0.8–3.3)
		AG&GG	21 (8.9%)	15 (7.2%)	20 (11%)			

*CHL, chlorthalidone; AML, amlodipine; LIS, lisinopril; CHD, coronary heart disease; HF, heart failure*.

*Dominant model was used for SNP which has any cell size less than 10 observations by collapsing minor allele homozygotes with heterozygotes which was compared to common allele homozygotes. Additive model coded common homozygotes as 0, heterozygotes as 1; rare homozygotes as 2; and assumed linearity (1-degree of freedom test)*.

With respect to the risk of HF, an association was observed among the African-American group for rs5051 when comparing chlorthalidone to amlodipine, rs2493129 when comparing chlorthalidone to lisinopril, and rs2493129 when comparing lisinopril to amlodipine with OR = 1.6, OR = 0.6, and OR = 2.0, respectively. A suggestive gene by treatment interaction was also observed among the Caucasian group at the rs2493129 locus with OR = 0.5 (*p* = 0.02) when comparing chlorthalidone to amlodipine. However, none of the tested models demonstrated statistical significance after the Bonferroni correction for multiple testing.

## Discussion

In this study, we evaluated whether *AGT* variants interact with antihypertensive treatments to effect the risk of CHD and HF during follow-up in GenHAT. Due to its high prevalence, the annual healthcare costs of hypertension are sharply on the rise (Heidenreich et al., 2011). Personalized antihypertensive treatment may benefit patients by maximizing blood pressure control and mitigating CVD morbidity and mortality resulting in reduced health care costs. Our results demonstrate that variation in the AGT gene might have a role in CHD and HF among hypertensive patients.

Other groups have considered the association between *AGT* polymorphisms and blood pressure response to antihypertensive agents. For instance, Frazier et al. (2004) found that the A allele of *AGT* G6A (rs5051) has a significant effect on systolic blood pressure reduction in response to 25 mg of hydrochlorothiazide (a thiazide diuretic) in African American women. Another study in 1447 hypertensive Chinese patients demonstrated that patients with the minor allele (A) of the *AGT* rs7079 SNP had a better blood pressure response to ACE inhibitors (benazepril) when compared to ACE users with the common allele (Su et al., 2007). Another group recently reported no difference in blood pressure response to hydrochlorothiazide associated with rs7079 (Huang et al., 2011). In sum, these studies suggest that variations in the *AGT* gene may have an influence on blood pressure response to antihypertensive therapies.

Other reports have found associations between SNPs in *AGT* and CVD events. For example, in a cohort study of 4097 hypertensive patients, ACE-inhibitor users with the minor allele (T) of M235T (rs699) had higher risk of having MI and stroke compared to non-users with the common allele (Schelleman et al., 2007). Another prospective study, the Thrombogenic Factor and Coronary Events (THROMBO) study, failed to show a significant association between M235T and recurrent coronary events in ACE inhibitor users (Goldenberg et al., 2006). Though these models are not directly comparable to our own, they support a role for the *AGT* gene in CVD risk during antihypertensive treatment.

Suggestive findings for rs11122576 (associated with a greater than two-fold risk of CHD) for two different treatment comparisons were observed in African Americans. Although these findings did not meet our significance criteria, they provide insight into genetic underpinnings of variation in response and outcomes. The pharmacogenetic effect of rs11122576 comparing chlorthalidone to amlodipine was in the opposite direction for Caucasians (OR = 0.6, *p* = 0.01). This may be due to differences in LD patterns in African Americans compared to Caucasians, but suggests the SNP could be tagging some important variant in both races. A recent study found a significant association of the minor allele of rs11122576 with CHD mortality in a cohort of 1186 CHD participants mostly of European ancestry-after 3-years of follow-up. The minor allele of rs1122576 was also associated with of higher risk of renal disease, type 2 diabetes, and higher body mass index in that study (Ellis et al., 2013). Similarly to the CHD results, there were more marginally significant associations among African Americans compared to Caucasians for the HF outcome, however none were close to statistical significance.

The GenHAT study is a large, ethnically diverse sample ancillary to a large clinical trial which facilitates pharmacogenetic studies among well-defined treatment groups with extensive adjudication of CVD outcomes. Despite this strength, our study also has some limitations. First, ALLHAT recruited hypertensive patients aged 55 years and older with a large proportion of diabetes mellitus (36.0%) and/or evidence of atherosclerotic cardiovascular disease (46.9%). Therefore, it may not be valid to generalize the findings of this study to healthier and younger populations. Second, the case-only design did not allow the evaluation of the main effects of treatment or genotype on the outcome (Khoury and Flanders, 1996). Third, we only had sufficient genotyped data for seven *AGT* SNPs, so this study cannot completely evaluate the antihypertensive pharmacogenetic effects of the *AGT* gene. Finally, and most importantly, we recognize most genomic findings are greatly strengthened by replication studies. However, due to specific drug regimens and SNP selections in GenHAT comparable study populations are difficult to find.

The findings reported here for antihypertensive treatment effects of seven *AGT* SNPs in 2253 CHD cases and 1728 HF cases suggest that a patient's *AGT* variants might help predict which hypertensive medication(s) can lower cardiovascular disease risk. Our results highlight rs11122576 for African American participants where the minor allele of rs11122576 was associated with over two-fold higher risk of CHD for chlorthalidone treatment compared to amlodipine treatment and lisinopril treatment compared to amlodipine treatment (*p* = 0.006 and *p* = 0.01, respectively). Although marginally significant, our results demonstrate biological relevance in a large, racially diverse population. These findings need to be investigated further and confirmed in independent sample of hypertensive patients.

### Conflict of interest statement

The authors declare that the research was conducted in the absence of any commercial or financial relationships that could be construed as a potential conflict of interest.
